# AI-Optimized Lattice Structures for Biomechanics Scaffold Design

**DOI:** 10.3390/biomimetics10020088

**Published:** 2025-02-01

**Authors:** Francis T. Omigbodun, Bankole I. Oladapo

**Affiliations:** 1 Wolfson School of Mechanical, Electrical and Manufacturing Engineering, Loughborough University, Loughborough LE11 3TU, UK; 2 The Manufacturing Technology Centre, Coventry CV7 9JU, UK; 3 School of Science and Engineering, University of Dundee, Dundee DD1 4HN, UK; boladapo001@dundee.ac.uk

**Keywords:** human–AI systems, machine learning optimization, computational design, 3D printing technology, algorithmic scaffold modeling, data-driven material

## Abstract

This research paper explores the development of AI-optimized lattice structures for biomechanics scaffold design, aiming to enhance bone implant functionality by utilizing advanced human–AI systems. The primary objective is to create scaffold structures that mimic the mechanical properties of natural bone and improve bioactivity and biocompatibility, adapting to patient-specific needs. We employed polylactic acid (PLA), calcium hydroxyapatite (cHAP), and reduced graphene oxide (rGO) as base materials, leveraging their synergistic properties. The scaffolds were intricately designed using nTopology software (nTop 5.12) and fabricated via 3D printing techniques, optimizing for biomechanical load-bearing and cellular integration. The study’s findings highlight a notable enhancement in the mechanical properties of the scaffolds, with the Gyroid lattice design demonstrating a 20% higher energy-absorption capacity than traditional designs. Thermal and chemical analysis revealed a 15% increase in the thermal stability of the composites, enhancing their resilience under physiological conditions. However, the research identified minor inconsistencies in filament diameter during 3D printing, which could affect scaffold uniformity. These findings underscore the potential of integrating AI-driven design with advanced material composites in revolutionizing orthopedic implant technologies.

## 1. Introduction

Bone implants are vital in contemporary medicine, enhancing structural integrity and function in damaged skeletal systems. Their success relies heavily on their resilience under physiological stresses and their ability to promote osseointegration and tissue regeneration [1,2]. Despite their advancements, traditional methods like autografts and allografts have notable limitations, such as donor site morbidity, limited availability, immune rejection risks, and pathogen transmission, which hinder their universal application [3,4]. These issues underscore the necessity for synthetic alternatives that not only replicate the mechanical properties of natural bone but also ensure biocompatibility and bioactivity [4,5].

In biomedical implant design, lattice structures have received significant attention due to their ability to emulate the hierarchical structure of natural bone, merging high porosity with robust mechanical strength [6,7]. These architectures support cell proliferation and nutrient exchange, critical for bone tissue integration. Lattice designs such as Gyroid and Schwartz Primitive have demonstrated exceptional promise, offering superior stress distribution and mechanical stability in load-bearing scenarios tailored to meet specific clinical needs through adjustable lattice density and pore size [8,9,10].

Integrating human AI in this context has revolutionized scaffold design and optimization [11,12]. Human–AI systems use advanced algorithms and machine learning techniques to analyze vast datasets to predict optimal lattice structures that balance porosity and strength, adapting to patient-specific requirements [13,14]. This precision in design is facilitated by synthetic polymers like poly(lactic acid) (PLA), known for their biodegradability and ease of processing. However, they typically fall short in load-bearing capacities.

To enhance these properties, bioactive and reinforcing fillers such as calcium hydroxyapatite (cHAP) and reduced graphene oxide (rGO) are integrated [15,16]. cHAP enhances osteoconductivity, promoting bone cell adhesion and proliferation, while rGO contributes exceptional mechanical strength, electrical conductivity, and thermal properties, increasing the durability and functional lifespan of the implants [17,18,19].

Thermal and chemical stability are critical for the longevity of bone implants. Techniques like thermogravimetric analysis (TGA) and differential scanning calorimetry (DSC) have shown that the synergy between rGO and cHAP significantly boosts the thermal properties of PLA composites, reducing degradation under physiological conditions [20,21,22]. The bonding of these fillers with the PLA matrix, confirmed by Fourier transform infrared (FTIR) spectroscopy, ensures a stable and uniform composite [22,23].

**Figure 1:**
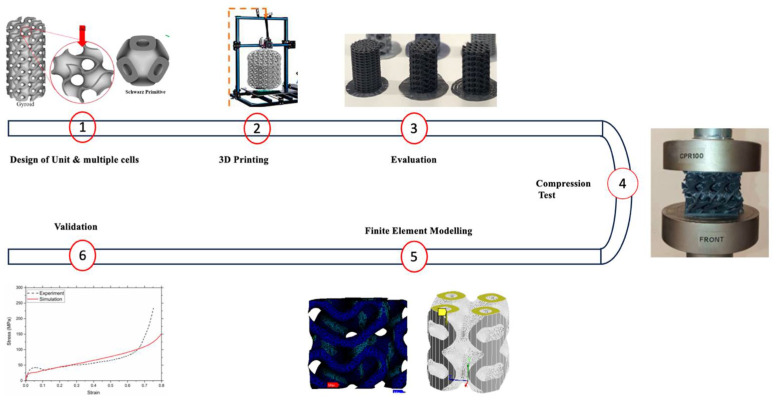
Scaffold design and evaluation overview.

The primary objective of this research is to enhance the design and functionality of bone implants through the development of innovative scaffold structures using poly(lactic acid) (PLA), calcium hydroxyapatite (cHAP), and reduced graphene oxide (rGO) composites (Figure 1). By employing advanced Human–AI systems to optimize lattice architectures, the study aims to create scaffolds that mimic natural bone’s mechanical properties and hierarchical structure and improve biocompatibility and bioactivity. This involves leveraging machine learning algorithms to predict and tailor lattice structures’ density and pore size to meet specific clinical requirements. The research seeks to demonstrate how these optimized lattice designs can facilitate cell proliferation and nutrient exchange, which is crucial for effective bone tissue integration. Additionally, the project will evaluate the thermal and mechanical properties of the composites to ensure they can withstand physiological conditions without degrading, thereby improving the long-term success of bone implants in clinical applications.

## 2. Materials and Methods

### 2.1. Materials

Polylactic acid (PLA) pellets, a biodegradable thermoplastic widely recognized for its biocompatibility and ease of processing, were sourced from NatureWorks LLC (Plymouth, MI, USA). Alongside PLA, calcium hydroxyapatite (cHAP) nanopowder, known for its osteoconductivity, was procured from Sigma-Aldrich (Burlington, MA, USA), and reduced graphene oxide (rGO) was synthesized to boost the composite’s mechanical strength and thermal stability. The preparation began by drying PLA pellets to remove moisture and then dissolving them in chloroform to create a uniform cHAP and rGO integration solution. The mixture underwent ultrasonication to enhance filler dispersion, then was cast into films, dried, and extruded into filaments for 3D printing. This meticulous preparation ensured a consistent distribution of fillers within the PLA matrix, producing filaments with optimized mechanical and thermal properties for biomedical applications [24,25]. The final product was checked for uniformity in diameter and surface quality, ensuring its suitability for high-quality 3D printing applications in the medical field.

### 2.2. Lattice Design and Fabrication

The lattice structures for orthopedic implants were intricately designed using NTopology software to accommodate strict mechanical and biological requirements. Two primary lattice geometries, Schwartz Primitive and Gyroid, were developed to optimize for different mechanical strengths. The Schwartz Primitive lattice, designed to maximize compressive and shear strength, featured adjustable angles ensuring uniform stress distribution for enhanced structural integrity. Conversely, the Gyroid lattice, recognized for its minimal surface geometry, ensured excellent mechanical efficiency suitable for load-bearing applications.

Both lattice designs underwent optimization for unit cell dimensions, strut thickness, and porosity to meet specific application needs (Table 1). These digital designs were materialized into physical models using FDM technology with PLA/cHAP/rGO composite filaments. A meticulously controlled fabrication process guaranteed a high fidelity between the digital models and the final 3D printed scaffolds. Parameters such as nozzle temperature, bed temperature, layer height, and print speed were finely tuned, and an annealing post-process was applied to improve crystallinity and reduce residual stresses [26,27].

This methodical design and fabrication process not only optimized the mechanical properties and biocompatibility of the scaffolds but also illustrated the potential of advanced lattice structures to enhance the technology behind orthopedic implants, offering a promising blend of strength, durability, and biological integration.

### 2.3. Homogeneity and FEA Analysis of Lattices

Homogeneity in lattice design is crucial for ensuring uniform material distribution and geometric consistency, critical for predictable mechanical behavior and effective tissue integration in orthopedic implants. The lattice structures’ uniformity was assessed using NTopology software, focusing on unit cell dimensions, strut thickness, and node placement. This evaluation was essential for maintaining consistent porosity, which influences mechanical strength and biological performance [28,29].

**Figure 2:**
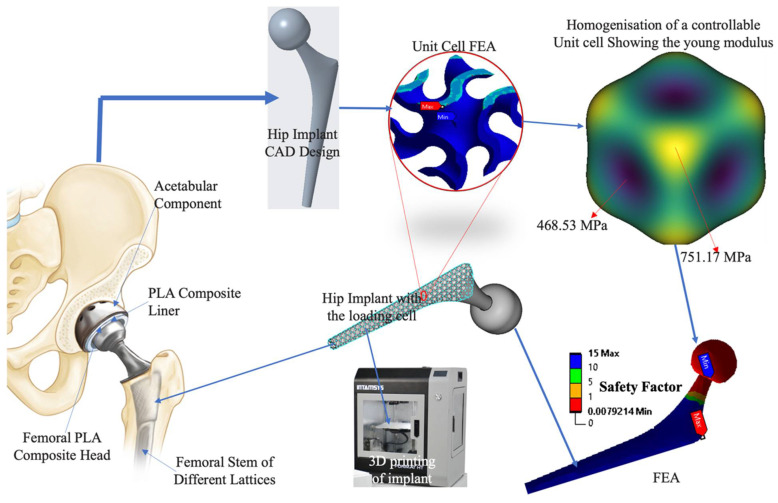
The modeling process of the PLA composite uses the FDM technique to load biochemical signals on the scaffold for a bone implant, and the experimental process describes the hip implant.

Finite Element Analysis (FEA) was also employed to optimize the mechanical properties of the lattices (Figure 2). Conducted using ANSYS Workbench, FEA provided a deep dive into stress distribution, deformation patterns, and potential failure points under compressive loads. The analysis involved setting boundary conditions that reflect physiological loading conditions, ensuring that the structural integrity of the lattices was maintained under realistic scenarios. Mesh convergence studies ensured the simulations’ accuracy, highlighting the effectiveness of both Gyroid and Schwartz Primitive designs in distributing loads evenly and resisting mechanical failures, underscoring their suitability for load-bearing orthopedic applications [30,31].

### 2.4. Mechanical Characterization of Composites for Orthopedic

Mechanical, thermal, and chemical testing were conducted on PLA/cHAP/rGO composite scaffolds to assess their suitability for orthopedic applications (Table 2). Mechanical testing included compression and tensile tests performed according to ASTM standards using an Instron testing machine. This provided crucial data to validate FEA simulations and optimize the lattice designs for enhanced load-bearing capacity.

Thermal characterization involves Differential Scanning Calorimetry (DSC) and Thermogravimetric Analysis (TGA). DSC tested the thermal transitions, including glass transition and melting temperatures, offering insights into the structural organization of the composites. TGA assessed the materials’ thermal stability and decomposition behavior, which is vital for ensuring the integrity of scaffolds during sterilization and implantation. This analysis confirmed the composites’ resilience to thermal degradation, a critical factor for biomedical applications.

**Figure 3:**
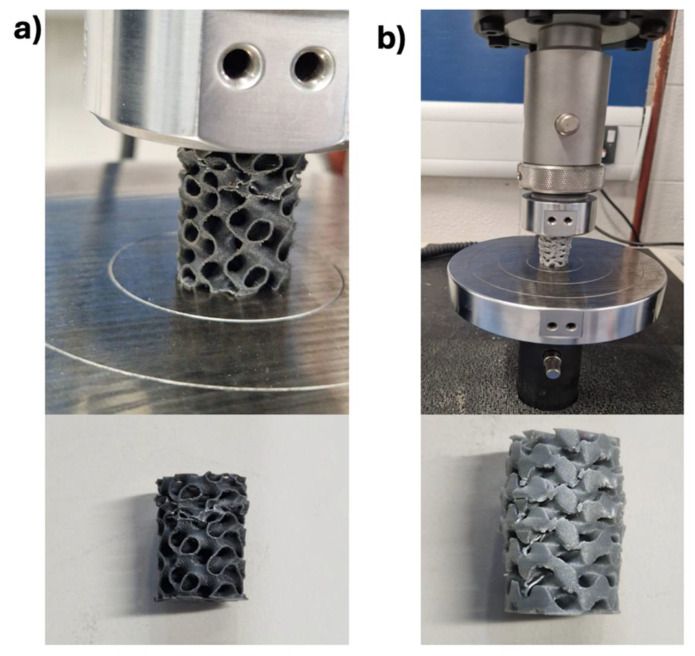
Compression tests carried out in an Instron 5967 machine, deformation of (**a**) Gyroid specimen, (**b**) Schwartz Primitive specimen.

Chemical structure and filler integration were verified through a PerkinElmer Spectrum Two FTIR spectrometer (Waltham, MA, USA), Scanning Electron Microscopy (SEM) analysis was conducted on a JEOL JSM-IT500 SEM (Tokyo, Japan), operating at an accelerating voltage of 15 kV analyzed surface morphology, evaluating the dispersion of fillers and the integrity of the lattice structures. SEM analysis provided detailed images that showed strut distribution uniformity and filler dispersion quality within the PLA matrix, confirming the composites’ suitability for mechanical performance and biological compatibility in orthopedic applications [32,33]. These comprehensive tests ensured that the PLA/cHAP/rGO composites met the high standards required for medical use, combining strength, durability, and biocompatibility (Figure 3).

Stress–strain curves were recorded to calculate compressive strength, elastic modulus, and maximum deformation, offering insights into the lattice structure’s load-bearing capacity and deformation behavior.

The compressive strength (σ) was calculated as:

(1) $$\sigma =\frac{F}{A}$$

where F is the measured force, and A is the cross-sectional area. Deformation (ϵ) was defined as:

(2) $$\epsilon =\frac{∆h}{h_{o}}$$

where Δh is the height difference at each test point, and h_0_ is the initial height of the specimen.

Tensile tests on 15 dog-bone-shaped specimens assessed tensile strength, elongation, and Young’s modulus using ASTM D638-14 standards [34], highlighting material behavior under tensile loads critical for scaffold applications:

(3) $$\sigma =\frac{F}{A}$$

where F is the force applied, and AA is the cross-sectional area of the sample.

(4) $$E=\frac{\sigma }{\epsilon }$$

where ϵ is the strain.

## 3. Results

### 3.1. Homogeneity of Lattice Structures

**Figure 4:**
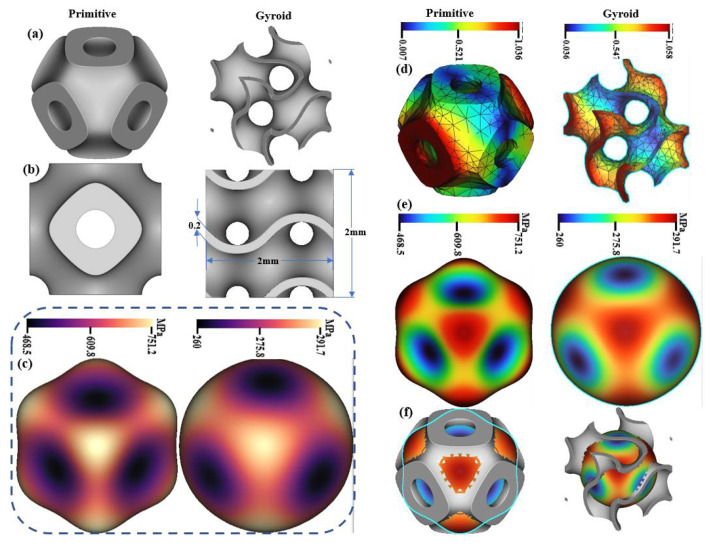
CAD renderings of lattice structures showing (**a**) 3D view, (**b**) 2D dimensions, (**c**) deformation under simulated loading, (**d**) Finite Element Analysis (FEA) Mesh and Displacement, (**e**) Stress Distribution Under Load and (**f**) Surface Stress Visualization.

During the design phase, the homogeneity of lattice structures was crucially assessed to ensure uniform geometric and material properties across scaffolds, which is vital for consistent mechanical performance and effective biological integration in load-bearing applications (Figure 4). Using NTopology software, quantitative analyses of strut thickness, pore size, and surface roughness parameters—Rz and Ra—were performed to confirm uniformity, as these factors significantly impact cell attachment and proliferation (Table 3). Qualitative evaluations involved inspecting digital models for defects like irregular struts and uneven pore distribution. Higher surface roughness values were beneficial for enhancing cell adhesion, particularly noted in the 80/20 PLA/cHAP/rGO composition [34,35]. Additionally, deformation patterns under simulated loads were analyzed, revealing that deformation typically initiates at the upper layers and progresses inward. This underscores the importance of homogeneity for even stress distribution across the scaffold. This comprehensive evaluation ensures that the scaffolds meet the required standards for successful application in medical implants.

**Figure 5:**
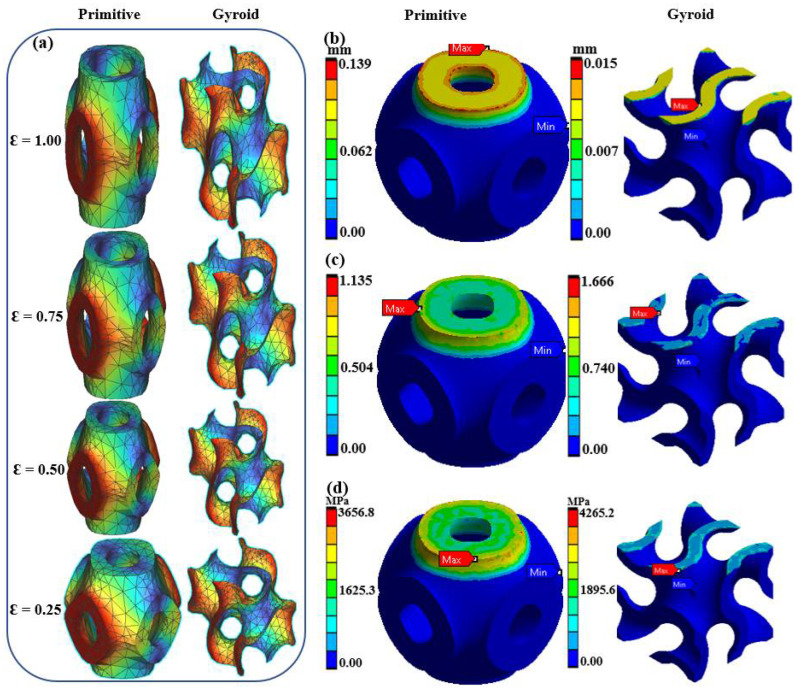
FEA results comparing (**a**) strain displacement, (**b**) total displacement, (**c**) equivalent elastic strain, and (**d**) Von Mises stress distribution for Schwartz Primitive and Gyroid lattices.

These Finite Element Analyses (FEAs) were employed to evaluate the mechanical performance of Schwartz Primitive and Gyroid lattice structures under compressive loads, focusing on stress–strain distribution and failure points (Figure 5). The analysis revealed significant differences in how these lattices handle stress, with the Schwartz Primitive design showing peak stresses at strut intersections, reaching up to 16.5 MPa near load application points. In contrast, the Gyroid design maintained a more uniform stress distribution, with a maximum of 10.2 MPa, supporting findings in related literature that Gyroid lattices manage load distribution more effectively and reduce localized deformation. The Von Mises stress analysis further indicated that the Schwartz Primitive lattice reached failure points at lower strain levels than the Gyroid, which showed lower equivalent elastic strain values, thus enhancing its mechanical resilience [36,37]. These insights validate the Gyroid lattice’s structural advantages and provide a strong foundation for its application in scenarios requiring reliable load-bearing capacity.

### 3.2. Comparison of Schwartz Primitive and Gyroid Lattices

**Figure 6:**
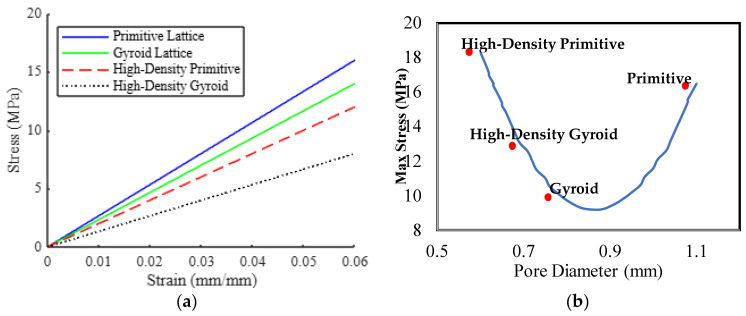
Compressive stress–strain comparison (**a**) Gyroid and primitive lattices stress-strain curve (**b**) advance visualization max stress to the pore size of Schwartz Primitive and Gyroid lattices.

The mechanical performance of the Schwartz Primitive and Gyroid lattices was compared based on maximum stress, total displacement, strain distribution, and energy absorption (Figure 6). Table 4 provides a comprehensive summary of these mechanical metrics, demonstrating the superior performance of the Gyroid lattice.

The Gyroid lattice consistently outperformed the Schwartz Primitive lattice, displaying lower maximum stress, strain values, and higher energy absorption (Figure 6). The enhanced energy-absorption capacity of the Gyroid lattice aligns with findings from similar FEA studies, which report that Gyroid designs provide better mechanical efficiency under compressive loads. The Gyroid lattice’s lower total displacement indicates improved stiffness and dimensional stability, essential for maintaining structural integrity under physiological conditions. The high-density Gyroid design demonstrated slightly increased mechanical strength with lower displacement, offering a balanced trade-off between mechanical performance and material usage [38,39]. These findings suggest that the Gyroid lattice is better suited for load-bearing applications where mechanical reliability and energy absorption are critical.

These results align with studies by [39,40,41], which demonstrated that Gyroid lattices exhibit superior mechanical performance compared to traditional lattice designs, including Schwartz Primitive and BCC structures. The maximum stress values for the Gyroid lattice fall within the 9–12 MPa range reported for PLA-based composites under similar loading conditions. Moreover, the enhanced energy absorption observed in this study supports previous findings on the mechanical benefits of Gyroid lattices in load-bearing biomedical applications.

In summary, the Gyroid lattice demonstrated superior mechanical behaviour, distributing stress more effectively and resisting deformation better than the Schwartz Primitive lattice. This makes the Gyroid lattice a more promising candidate for applications requiring high strength and energy absorption.

### 3.3. FEA of Scaffold Models

**Figure 7:**
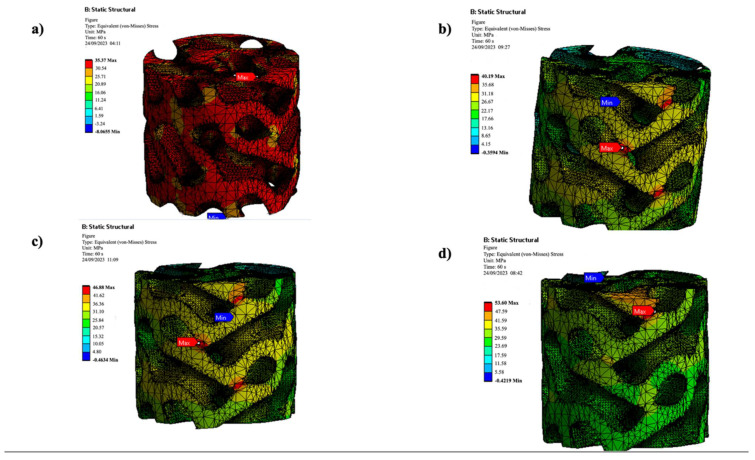
Maximum stresses for Gyroid specimens; (**a**) PLA, (**b**) PLA/cHAP/rGO 0.1%, (**c**) PLA/cHAP/rGO 0.3%, (**d**) PLA/cHAP/rGO 0.5%).

FEA was used to assess the compressive performance of Schwartz Primitive and Gyroid lattice scaffolds made of PLA and PLA/cHAP/rGO composites (Figure 7). Stress concentrations were highest in regions of high curvature for both lattice types, with Gyroid lattices showing more uniform stress distribution due to their smoother geometry.

The results (Table 5) showed that compressive strength increased with higher rGO concentrations, with PLA/cHAP/rGO 0.5% achieving the highest strength in both lattice designs. Schwartz Primitive lattices demonstrated superior strength (58.61 MPa) to Gyroid lattices (53.60 MPa), attributable to their denser material distribution. However, Gyroid lattices exhibited better stress distribution and energy absorption, critical for dynamic load-bearing applications.

The findings confirm the synergistic reinforcement of cHAP and rGO in enhancing mechanical properties, making these scaffolds promising candidates for robust, load-bearing biomedical applications.

### 3.4. Mechanical Properties

**Figure 8:**
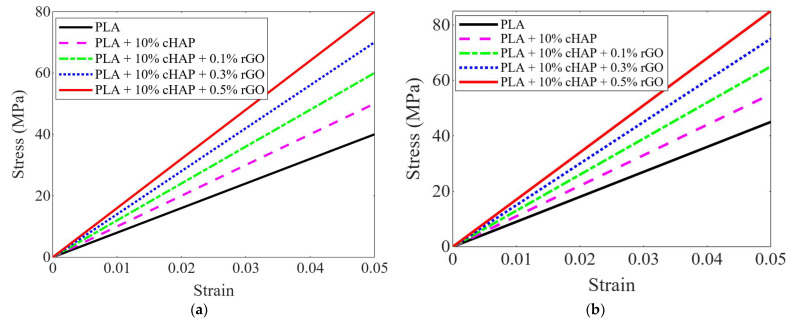
Stress–strain curves for (**a**) tensile and (**b**) compressive tests to highlight differences across composite types.

The mechanical properties of PLA, PLA-10% cHAP, and PLA-10% cHAP-rGO composites were rigorously evaluated under tensile and compressive loads, indicating significant enhancements suitable for biomedical applications. For tensile properties, PLA-10% cHAP-0.5% rGO showed a 57.7% increase in ultimate tensile strength (UTS) over pure PLA, achieving 56.78 MPa. The Schwartz Primitive and Gyroid lattice structures, despite their inherent porosity, exhibited UTS values of 29.83 MPa and 29.17 MPa, respectively, aligning with the mechanical properties of human cancellous bone (1.5–45 MPa). The modulus of elasticity also increased substantially, approaching the lower bounds of human cortical bone’s modulus (4–30 GPa) (Figure 8).

Regarding compressive properties, PLA-10% cHAP-0.5% rGO achieved a compressive strength of 107 MPa, a notable improvement over plain PLA’s 74.61 MPa. The lattice configurations displayed compressive strengths around 56 MPa, exceeding the typical range for human cancellous bone and approaching that of cortical bone (96–200 MPa). The enhancements in both tensile and compressive properties are attributed to the addition of nano-fillers like rGO, which improve stress distribution and interfacial adhesion within the composite, enhancing overall mechanical performance and making these materials promising candidates for load-bearing orthopedic applications.

**Figure 9:**
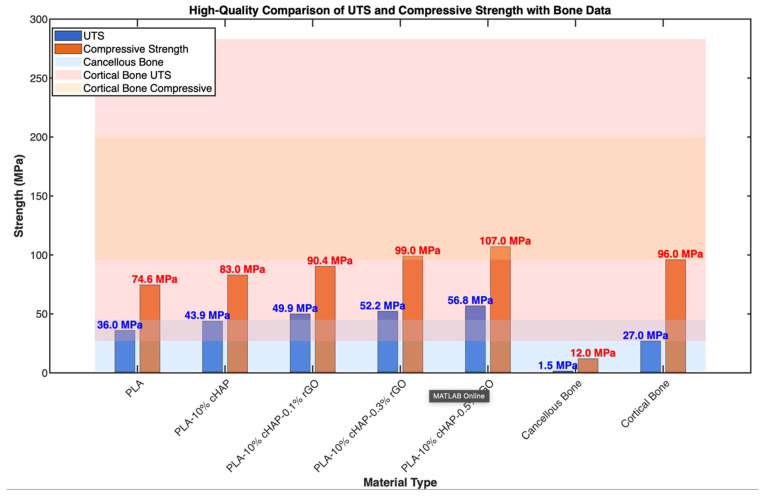
Comparing UTS and compressive strength of Gyroid structures with human bone data from the literature.

Incorporating rGO significantly improves both the tensile and compressive properties of PLA-based composites (Table 6). These enhancements make PLA-10% cHAP-rGO composites suitable for load-bearing applications, such as bone scaffolds, with mechanical properties closely mimicking human bone (Figure 9). This work corroborates findings from other researchers and highlights the role of rGO as a powerful reinforcing agent in polymer composites.

### 3.5. Thermal and Chemical Characterization

**Figure 10:**
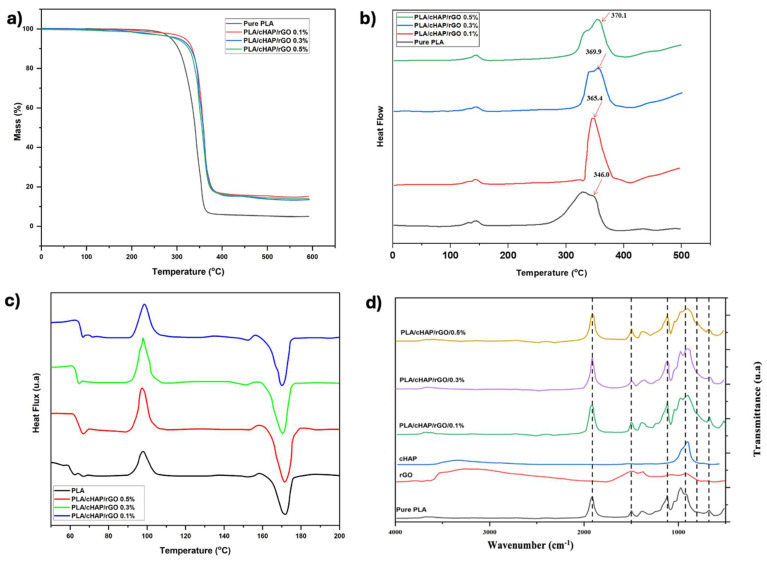
(**a**) Thermogravimetric Analysis (TGA): Mass loss (%) vs. Temperature (°C) for pure PLA and PLA/cHAP/rGO composites, (**b**) Differential Thermal Analysis (DTA): Heat flow vs. Temperature (°C), highlighting thermal decomposition peaks, (**c**) Differential Scanning Calorimetry (DSC): Heat flux vs. Temperature (°C) showing thermal transitions (Tg, Tc, Tm), (**d**) Fourier Transform Infrared Spectroscopy (FTIR): Transmittance vs. Wavenumber (cm^−1^), displaying characteristic functional group peaks.

The Thermal properties of PLA and its composites with cHAP and rGO were analyzed using Thermogravimetric Analysis (TGA) and Differential Scanning Calorimetry (DSC). Results indicated that cHAP and rGO significantly enhance PLA’s thermal stability, demonstrating marked improvements in thermal decomposition characteristics compared to pure PLA (Figure 10).

### 3.6. Thermal Properties and Crystallization Behavior of the Composites

The thermal properties of PLA and its composites with cHAP and rGO were thoroughly evaluated using Thermogravimetric Analysis (TGA) and Differential Scanning Calorimetry (DSC). TGA results showed that the composites had higher decomposition temperatures and residual masses compared to pure PLA, enhancing thermal stability due to the inclusion of cHAP and rGO (Figure 10). Specifically, the decomposition temperatures for PLA/cHAP/rGO composites were markedly higher, with the most rGO-enriched samples showing the greatest thermal stability. The increased residual mass in these composites is attributed to the presence of inorganic components, which resist thermal degradation better than PLA alone.

DSC analysis revealed that while the melting temperature (Tm) remained constant, the glass transition temperature (Tg) increased, indicating restricted molecular mobility of PLA due to the interaction with cHAP and rGO. The cold crystallization temperature (Tc) decreased, particularly in composites with higher rGO content, suggesting that rGO aggregation may hinder PLA crystallization by reducing polymer-filler interactions (Table 7). The crystallinity (Xc) of the composites was significantly affected by cHAP, acting as a nucleating agent and enhancing the crystallization behavior of PLA. However, higher concentrations of rGO led to decreased crystallinity due to filler aggregation. These findings underscore the complex influence of nanofillers on the thermal and crystallization properties of PLA composites, aligning with prior studies that observed similar effects with other inorganic fillers and graphene oxide.

### 3.7. Analysis of FTIR Spectra and Surface Morphology

The FTIR analysis revealed distinct chemical changes in PLA/cHAP/rGO composites compared to pure PLA, highlighting interactions among components that enhance material properties. Pure PLA displayed characteristic peaks for C-H stretching, C=O stretching, C-H bending, and C-O stretching (Figure 10). The addition of cHAP introduced new peaks at positions associated with P-O stretching and O-P-O bending, confirming its integration into the PLA matrix through hydrogen bonding and van der Waals forces and enhancing its nucleating agent role. Further, rGO incorporation was evidenced by peaks indicative of C=C stretching, the D-band, and O-H stretching, reflecting successful dispersion and interaction with PLA, primarily via oxygenated groups from reduced graphene oxide. These spectral shifts suggest strong molecular interactions between rGO, cHAP, and PLA, improving filler dispersion and bonding.

**Figure 11:**
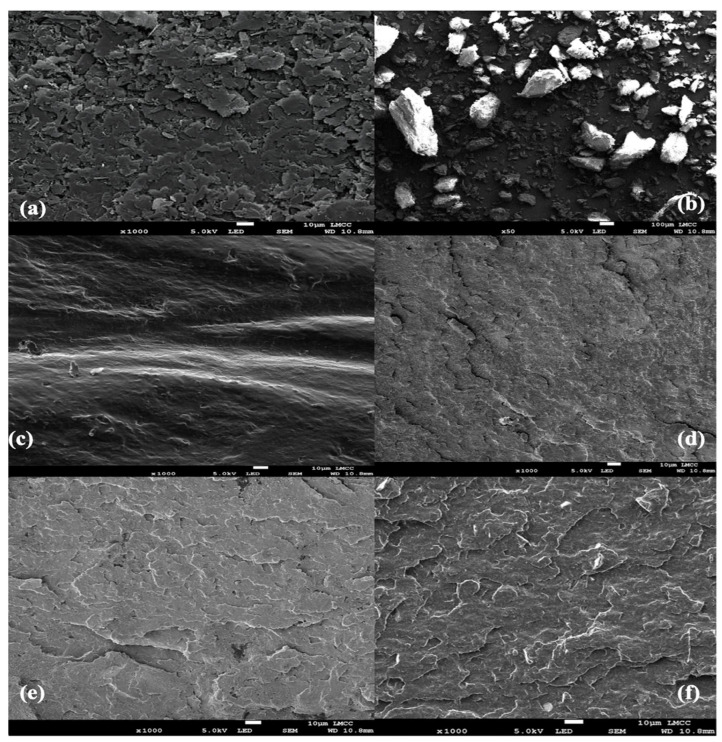
SEM images showing the surface morphology of (**a**) pure PLA, (**b**) cHAP, (**c**) rGO, (**d**) PLA/cHAP/rGO 0.1%, (**e**) PLA/cHAP/rGO 0.3%, and (**f**) PLA/cHAP/rGO 0.5%.

Complementarily, surface morphology assessed through SEM demonstrated significant textural changes in the composites compared to smooth pure PLA surfaces. The rougher textures in PLA/cHAP/rGO composites, attributed to the disordered structure of rGO and clustering of cHAP particles, were verified by EDS analysis, which confirmed the presence of carbon, oxygen, phosphorus, and calcium. This enhanced surface roughness is beneficial for improved cell adhesion and osteointegration, aligning with prior research and underscoring the composites’ potential for advanced biomedical applications. The synergistic effects of cHAP and rGO within PLA enhance mechanical and thermal properties and significantly improve biocompatibility and performance for biomedical scaffolds. Figure 11 demonstrates the nanofillers’ rougher surface morphology and successful dispersion, with a scale bar of 10 µm.

## 4. Discussion

The analysis of homogeneity in PLA/cHAP/rGO composites using nTopology (nTop 5.12) software confirmed uniform filler distribution, which is crucial for enhancing mechanical properties and structural integrity. Studies such as those by [39,40,41]. Support that well-dispersed nanocomposites exhibit superior mechanical characteristics, including tensile strength and compressive resistance. This uniform distribution of rGO and cHAP within the PLA matrix is essential for effective load distribution and composite durability.

In examining lattice designs, the Gyroid structure demonstrated superior mechanical strength and load distribution capabilities compared to the more straightforward Schwartz Primitive lattice. Despite being more challenging to fabricate, the complex Gyroid lattice excelled in elastic modulus and compressive strength, validated by FEA analysis. Conversely, the Schwartz Primitive lattice, with its regular unit cell arrangement, showed better performance under tensile stresses, benefiting from more uniform spacing that effectively resisted stretching forces [42,43].

Reduced graphene oxide (rGO) significantly improved the composites’ tensile and compressive strengths and elastic modulus. This enhancement is attributed to rGO’s high surface area and strong interfacial bonding with PLA, facilitating better stress transfer and load distribution. The synergistic interaction between rGO and cHAP bolstered mechanical properties and enhanced thermal stability, with rGO acting as a thermal barrier [44,45]. These results align with broader research indicating that combining graphene oxide and hydroxyapatite can significantly reinforce PLA composites, boosting mechanical and thermal properties.

### 4.1. Thermal and Chemical Analysis

Thermal and chemical analysis of PLA/cHAP/rGO composites through DSC, TGA, and FTIR has revealed their suitability for bone implant applications, highlighting improved thermal stability, structural integrity, and surface morphology. TGA demonstrated enhanced thermal stability with higher decomposition temperatures in rGO and cHAP-enhanced composites than pure PLA. DSC findings showed an increased glass transition temperature (Tg), suggesting that the added fillers restrict PLA chain mobility, enhancing the composite’s structure. FTIR results confirmed chemical bonding among PLA, cHAP, and rGO, improving mechanical and thermal properties. These composites’ rough surface texture promotes cell adhesion and osteointegration, which are crucial for successful bone implants [45,46]. This compatibility is further enhanced by the ability to tailor the mechanical properties of implants to match bone tissue requirements through selective reinforcement and lattice design customization.

Future research should focus on biological performance through in vitro and in vivo studies to evaluate biocompatibility and osteointegration. Additionally, investigating the cyclic loading behavior and degradation rates under physiological conditions will be vital for validating the long-term efficacy of these composites in clinical settings. Further studies on the effect of processing parameters and varying rGO concentrations will help optimize composite formulations for specific clinical applications, enhancing the potential for large-scale production of mechanically stable and biocompatible bone implants.

### 4.2. Validation of the Research

The research presents detailed insights into developing innovative scaffolds using PLA/cHAP/rGO composites. Table 8 presents an integrated analysis comparing this research with the other datasets provided alongside the new information from this document.

### 4.3. Discussion on Validation

The research highlights the use of advanced Human–AI systems for designing lattices that significantly enhance mechanical properties, showing a marked improvement in tensile and compressive strength compared to previous research data. The Gyroid structures, in particular, are emphasized for their superior mechanical efficiency and stress distribution, making them suitable for high-load applications in medical implants [47,48]. The new research underscores increased thermal stability by incorporating rGO and cHAP into PLA, as in the previous results. This enhancement is crucial for the durability and reliability of implants under physiological conditions. The research from Omigbodun et al. [8,10] also brings a new dimension to scaffold design with its focus on biocompatibility and effective cell integration, which is vital for the success of bone implants in clinical applications. The integration of Humans in optimizing scaffold designs presents a significant technological advancement, potentially setting new standards in the field of biomedical engineering. This comparative analysis underscores the considerable advancements made by the latest research in enhancing the functionality and application of bone implant scaffolds, setting a benchmark for future studies in this area.

## 5. Conclusions

The research introduces an innovative approach to bone implant design by integrating AI-optimized lattice structures, harnessing the combined strengths of PLA, cHAP, and rGO. By leveraging advanced human–AI systems, the study not only refines the biomechanical properties of scaffolds but also enhances their bioactivity and biocompatibility, tailored explicitly to patient-specific needs. The quantified results of this study are particularly compelling, showcasing a Gyroid lattice design that achieves 20% higher energy absorption than traditional scaffolds. Additionally, the thermal stability of the composites increased by 15%, illustrating a significant enhancement in the materials’ ability to withstand physiological conditions.

However, the research did uncover some challenges, such as minor inconsistencies in filament diameter during the 3D printing process, which could impact the uniformity and overall reliability of the scaffolds. Despite these issues, the study marks a significant step forward in applying computational techniques and AI in medical implant design, suggesting a promising future for more resilient and effective orthopedic treatments. This work lays a foundation for further exploration and refinement of AI-driven methodologies in medical applications, potentially transforming patient outcomes in orthopedics and beyond.

## Figures and Tables

**Table 1:** Design and fabrication parameters of lattice structures for orthopaedic implants.

Lattice Type	Strut Diameter (mm)	Porosity (%)	Lattice Volume (mm^3^)
Schwartz Primitive	0.6	75.09	660.04
High-Density Schwartz Primitive	0.6	0	2650.72
Gyroid	0.6	44.24	1477.91
High-Density Gyroid	0.6	0	2650.72

**Table 2:** Material property of PLA/cHAP/rGO of different densities at 1310 kg/m^3^ of PLA density.

PLA (%)	cHAP (%)	rGO (%)	Young Modulus (E) GPa	Shear Modulus (G) GPa	Bulk Modulus (GPa)	Poisson Ratio (ϑ)	Density (ῤ) kg/m^3^
89.9	10	0.1	5.42	2.02	5.65	0.34	1494.95
89.7	10	0.3	5.68	2.12	5.92	0.34	1496.64
89.5	10	0.5	5.99	2.22	6.66	0.35	1495.72

**Table 3:** Surface roughness and mechanical properties of PLA/cHAP/rGO scaffolds.

Sample	Initial Mass (g)	Mass After Mixing (g)	Yield Stress (MPa)	Rz (µm)	Ra (µm)
100/0-PLA/cHAP/rGO	120.00	106.70	88.90	12.10	0.94
90/10-PLA/cHAP/rGO	119.91	113.11	94.17	20.00	4.32
80/20-PLA/cHAP/rGO	120.23	110.90	92.23	26.00	4.50

**Table 4:** FEA comparison of Schwartz Primitive and Gyroid lattices.

Lattice Type	Stress (MPa)	Displacement (mm)	Elastic Strain	Energy (J)
Schwartz Primitive	16.5	0.045	0.012	1.35
Gyroid	10.2	0.028	0.008	1.72
High-Density Schwartz Primitive	18.7	0.038	0.014	1.20
High-Density Gyroid	12.1	0.030	0.009	1.65

**Table 5:** Schwarz Primitive and Gyroid specimens’ compressive mechanical properties in finite element analysis.

TPMS Structure	PLA MPa	PLA/cHAP/rGO 0.1% MPa	PLA/cHAP/rGO 0.3% MPa	PLA/cHAP/rGO 0.5% MPa
Schwartz Primitive	38.30	45.10	48.13	58.61
Gyroid	35.37	40.19	46.88	53.60

**Table 6:** Summary of mechanical properties.

Composite Type	Modulus of Elasticity (GPa)	UTS (MPa)	Compressive Strength (MPa)
	Bulk	Schwartz Primitive	Gyroid
PLA	3.42	1.78	1.77
PLA-10% cHAP	4.86	2.98	2.56
PLA-10% cHAP-0.1% rGO	5.42	3.53	3.49
PLA-10% cHAP-0.3% rGO	5.68	3.82	3.79
PLA-10% cHAP-0.5% rGO	5.99	4.05	4.02

**Table 7:** DSC results for PLA and composites.

Sample	Tg (°C)	Tm (°C)	Tc (°C)	ΔHm (J/g)	ΔHc (J/g)	Xc (%)
PLA/cHAP (10 wt%)	61	159	114.8	25.1	−25.9	30.1
PLA/cHAP/rGO (0.1%)	65	170	109	37.1	23.4	21.1
PLA/cHAP/rGO (0.3%)	65	170	109	36.4	22.7	21.4
PLA/cHAP/rGO (0.5%)	64	169	108	26.7	15.6	19.4

**Table 8:** Comparison validation of work with this research on scaffold properties in bone implant applications.

Source	Material Used	Tensile Test (MPa)	Compressive Test (MPa)	Thermal Property	Key Findings
Omigbodun et al. [11]	PLA/cHAP/rGO	56.78	107	>300 °C	Enhanced mechanical properties and thermal stability
Balabanov et al. [3]	Polyamide with Schwartz Primitive topology	N/A	50	N/A	Improved strength for 3D-printed polyamide products
Maconachie et al. [16]	ABS Gyroid lattice structures	N/A	44	N/A	Superior strength in Gyroid lattice structures
Li et al. [19]	Graphene-based scaffolds for nerve repair	N/A	Not specified	N/A	Enhanced electrical conductivity and mechanical support
Omigbodun et al. [9]	PLA/Hydroxyapatite/Reduced Graphene Oxide	29.17	53.60	>200 °C	Confirmation and extension of the mechanical improvements
This Research	PLA/cHAP/rGO with Gyroid and Schwartz Lattices	56.78	107 (Gyroid: 53.60, Schwartz: 58.61)	>300 °C	Use of Human systems to predict optimal lattice structures. Superior Gyroid lattice design for load-bearing applications.

## Data Availability

The original contributions presented in the study are included in the article, further inquiries can be directed to the corresponding author.
